# Equine Histoplasmosis in Ethiopia: Phylogenetic Analysis by Sequencing of the Internal Transcribed Spacer Region of rRNA Genes

**DOI:** 10.3389/fcimb.2022.789157

**Published:** 2022-07-08

**Authors:** Gobena Ameni, Alebachew Messele Kebede, Aboma Zewude, Musse Girma Abdulla, Rahel Asfaw, Mesfin Mamo Gobena, Martina Kyalo, Francesca Stomeo, Balako Gumi, Teshale Sori

**Affiliations:** ^1^ Aklilu Lemma Institute of Pathobiology, Addis Ababa University, Addis Ababa, Ethiopia; ^2^ Department of Veterinary Medicine, College of Food and Agriculture, United Arab Emirates University, Al Ain, United Arab Emirates; ^3^ College of Veterinary Medicine and Agriculture, Addis Ababa University, Debre Zeit, Ethiopia; ^4^ Capacity Building, Biosciences for Eastern and Central Africa-International Livestock Research Institute (BecA-ILRI) Hub, Nairobi, Kenya

**Keywords:** *Histoplasma capsulatum*, internal transcribed spacer region, phylogenetic analysis, equine histoplasmosis, Sanger Sequencing

## Abstract

Equine histoplasmosis commonly known as epizootic lymphangitis (EL) is a neglected granulomatous disease of equine that is endemic to Ethiopia. It is caused by *Histoplasma capsulatum* variety *farciminosum*, a dimorphic fungus that is closely related to *H. capsulatum* variety c*apsulatum.* The objective of this study was to undertake a phylogenetic analysis of *H. capsulatum* isolated from EL cases of horses in central Ethiopia and evaluate their relationship with *H. capsulatum* isolates in other countries and/or clades using the internal transcribed spacer (ITS) region of rRNA genes. Clinical and mycological examinations, DNA extraction, polymerase chain reaction (PCR), Sanger sequencing, and phylogenetic analysis were used for undertaking this study. Additionally, sequence data of *Histoplasma* isolates were retrieved from GenBank and included for a comprehensive phylogenetic analysis. A total of 390 horses were screened for EL and 97 were positive clinically while *H. capsulatum* was isolated from 60 horses and further confirmed with PCR, of which 54 were sequenced. BLAST analysis of these 54 isolates identified 29 *H. capsulatum* isolates and 14 isolates from other fungal genera while the remaining 11 samples were deemed insufficient for further downstream analysis. The phylogenetic analysis identified five clades, namely, African, Eurasian, North American 1 and 2, and Latin American A and B. The Ethiopian isolates were closely aggregated with isolates of the Latin American A and Eurasian clades, whereas being distantly related to isolates from North American 1 and 2 clades as well as Latin American B clade. This study highlights the possible origins and transmission routes of Histoplasmosis in Ethiopia.

## Introduction

Equine histoplasmosis is caused by *H. capsulatum* var. *farciminosum* which is a dimorphic fungus that is closely related to *Histoplasma capsulatum* variety *capsulatum* (*H. capsulatum* var. *capsulatum*), which is a causative agent of histoplasmosis in humans. *H. capsulatum* var. *farciminosum* has mycelia and yeast forms; the mycelial form lives in the soil in hot and humid areas while the yeast form is found in the lesions of infected equids. The zoonotic significance of *H. capsulatum* var. *farciminosum* has not been well investigated. However, there were reports of sporadic cases of humans that were not fully established by rigorous laboratory investigations (Al-Ani, 1999). Nonetheless, the World Organization for Animal Health (OIE, 2018) recommends that all laboratory procedures involving *H. capsulatum* var. *farciminosum* should be conducted with appropriate biosafety and containment procedures.

Equine histoplasmosis, commonly known as epizootic lymphangitis (EL), is a contagious chronic disease of equines characterized clinically by a suppurative pyogranulomatous dermatitis and lymphangitis at the early stage, which later becomes ulcerate as the disease progresses (Singh, 1965; Radostits, 1994). In equids, EL can appear in cutaneous, ocular, or/and respiratory forms (Singh, 1965; Ameni, 2007). The cutaneous form occurs as a result of direct or indirect contact between traumatized skin and infected and/or contaminated substances. The conjunctival form is assumed to be transmitted by flies of the *Musca* or *Stomoxys* genera (Singh, 1965), while inhalation of the causative agent leads to the development pulmonary form.

Epidemiologically, EL occurs more frequently in tropical and subtropical regions than in temperate regions, which is due to the favorable environmental condition for the survival of the mycelial form of *H. capsulatum* var. *farciminosum*. It is endemic in north, east, and northeast Africa and in countries bordering the Mediterranean Sea, India, Pakistan, East Africa, and Japan (Selim et al., 1985; Guérin et al., 1992; OIE, 2008). However, there is scarcity of literature on the epidemiology of EL in the equine population of these countries except an indication of their endemicity to the disease. Furthermore, there is little or no information on the epidemiology of human histoplasmosis in the tropical and subtropical regions of the world although the climatic condition is conducive for the replication of *Histoplasma*. Additionally, little effort was made on the isolation and characterization of *H. capsulatum* varieties in general and *H. capsulatum* var. *farciminosum* in particular. Most of the studies conducted so far were performed on human histoplasmosis and its causative agents, i.e., *H. capsulatum* var. *capsulatum* and *H. capsulatum* var. *duboisii*. Hence, studies are needed on the epidemiology of equine histoplasmosis and *H. capsulatum* var. *farciminosum*. A study conducted in Ethiopia between January 2003 and June 2004 on 19,082 carthorses at 28 towns reported a prevalence of 18.8% indicating the widespread occurrence of the disease in Ethiopia (Ameni, 2006). In addition to affecting horses, the disease also affects mules, and a prevalence of 21% was recorded in 309 cart mules in western Ethiopia (Ameni and Terefe, 2004). The infection in mules was observed to be highly associated with tick infestation (Guérin et al., 1992; Ameni and Terefe, 2004).

On top of its economic impact, because of its chronic and debilitating nature, EL is characterized by causing severe welfare problem to equines. The disease poorly responds to treatment. Moreover, EL requires a longer duration, which is not attractive for the owners of equines.

Azoles and amphotericin B are used for the treatment of histoplasmosis in humans and animals in developed countries. However, amphotericin B could not be used in developing countries where EL is most common for its cost (CFSPH. CFSPH, 2019). Instead, EL is treated with cheap drugs such as intravenous sodium iodine or oral potassium iodide, sometimes in conjunction with topical iodide applied to lesions and/or surgical excision. However, iodide has limited efficacy in moderate to severe cases (CFSPH. CFSPH, 2019). Hence, control by vaccination could be an alternative method that requires for the development of a new vaccine. To this effect, identifying the strains of *H. capsulatum* var. *farciminosum* that infect the equine population would contribute to designing of the potential vaccine candidates. The objective of this study was to undertake the phylogenetic analysis of *H. capsulatum* isolated from EL cases of horses in central Ethiopia and evaluate the relationship of the Ethiopian isolates with the isolates from other countries on the basis of the ITS region of rRNA genes.

## Materials and Methods

### Study Animals and Sampling

The screening of the horses for EL was conducted in central Ethiopia using clinical examination. Accordingly, 390 carthorses were examined for the characteristic clinical lesions of EL. The visible parts of the body of each horse were inspected for the presence of nodules and/or ulcers caused by EL. Inspection was made for the cutaneous, ocular, and respiratory forms of the disease. In addition, the contents of the nodular lesions were aspirated with needles and syringes and smeared on glass slides for microscopic examination. Thereafter, the slides were stained with Giemsa stain and then examined at ×100 using oil immersion for the typical yeast form of the organism, which appears as ovoid to globose in shape (Buxton and Fraser, 1977).

### Isolation of H. *capsulatum* var. *farciminosum*

The aspirates of the nodular lesions were cultured on Sabouraud Dextrose Agar enriched with 2.5% glycerol for the isolation of the mycelial form while Brain Heart Infusion Agar was used for the isolation of the yeast form. Chloramphenicol (0.5 g/l) was added to each of these two media to avoid the growth of bacterial contaminants. After inoculation of the samples onto the media, incubation was made at 26°C for 8 weeks for the isolation of the mycelial form while the isolation of the yeast form was made at 37°C for 8 weeks. The growth of dry, gray-white, granular wrinkled colonies within 4–8 weeks was considered as positive for the growth. A differentiation between the colonies of the two forms was made by Giemsa staining (OIE, 2008). As mycelial colonies were abundant, they were used for the extraction of DNA for sequencing.

### DNA Extraction and Amplification

The mycelial colonies of 60 culture positive samples were harvested and used for the extraction of DNA for subsequent molecular analysis. DNA extraction was done using the cetyltrimethylammonium bromide (CTAB) method (Mace et al., 2003). The concentration of the DNA was estimated using the NanoDrop ND 1000 spectrophotometer (Thermo Scientific, Wilmington, DE, USA) while the quality of the DNA was evaluated using GelRed^®^-based 1% gel electrophoresis. A pair of primers (ITS1 and ITS4) (8-11 Munpyeongseo-ro, Daedeok-gu Daejeon, 34302, Republic of Korea) was used for PCR reactions. The primers used were ITS1 (forward primer) with a sequence of TCC GTA GGT GAA CCT GCG G and ITS4 (reverse primer) with a sequence of TCC TCC GCT TAT TGA TAT GC (White et al., 1990). The final concentration of each of the two primers was 0.1 μM/μl. Amplification was carried using a 50-μl reaction volume containing 40 ng of DNA template, PCR buffer containing 50 mM KCl, 10 mM Tris–HCl (pH 8.3), and 3 mM MgCl**_2_**, 0.2 mM of each deoxynucleoside triphosphate (dNTPs), 25 pmol of each primer, and 1 U of *Taq* DNA polymerase. PCR was performed in the thermal cycler (Eppendorf Mastercycler) programmed for the first denaturation at 95°C for 5 min followed by 35 cycles of the succeeding step denaturation at 95°C for 45 s, annealing at 58°C for 1 min and extension at 72°C for 1 min, and a final extension period of 72°C of 7 min. The PCR products were electrophoresed in 1.8% agarose (Sigma Chemical Co., St. Louis, MO) using GelRed^®^ at 100 V for 45 min, and the bands were visualized with a UV transilluminator.

### Purification of the Amplicon for Sequencing

Amplicons were purified using QIAquick^®^ PCR Purification Kit (Qiagen^®^, Hilden, Germany) according to the manufacturer’s protocols. Following the purification, the concentration and purity of DNA were measured using the NanoDrop spectrophotometer while its integrity was examined using GelRed-based gel electrophoresis. Pure DNA samples with 20 ng/μl or greater concentrations with distinct bands on agarose gel were used for sequencing. Sequencing was conducted at Bioneer Corporation (8-11 Munpyeongseo-ro, Daedeok-gu Daejeon, 34302, Republic of Korea) using the Sanger capillary sequencing method. To this effect, purified PCR products of 60 samples, a positive control, and a pair primer (ITS1 and ITS4) were submitted to Bioneer Corporation through the Biosciences Eastern and Central Africa-International Livestock Research Institute (BecA-ILRI, Kenya) Hub and the sequence data were received.

### Sequence Curation

The trace files of all samples were checked for quality and subjected to trimming using a quality score of 0.05 with ambiguous nucleotides, and thereafter the trimmed sequences were saved for further subsequent analysis. Sequence curation was performed using SeqMan Pro 15 DNASTAR Lasergene 15. The forward and reverse sequences of each sample were assembled using the assembly parameters, which included a minimum aligned read length of 50, medium alignment stringency, and conflict votes of A, C, G, and T. Full contigs and consensus sequences were created using these assembly parameters. Trace files which did not fulfil these requirements and those with poor quality to obtain the full-length data were removed from downstream analysis.

### Phylogenetic Analysis

For the analysis of the phylogenetic relationship among worldwide isolates, the ITS1-5.8s-ITS2 region sequences of *H. capsulatum* were retrieved from the National Center for Biotechnology Information (NCBI, nig.gov) database. Retrieved GenBank sequences corresponding to *H. capsulatum* were aligned with nucleotide sequences of the isolates of the present study using the Clustal W program. Nucleotide substitutions of the best-fit model for each locus were statistically selected using jModelTest 2.0. Phylogenetic relationships were then estimated using Bayesian methods as implemented in MrBayes v3.2.6. A Tamura-Nei model + gamma model was used as it was assumed to best fit the data. Bayesian support for nodes was inferred in MrBayes using 42 × 106 Markov Chain Monte Carlo (MCMC) steps, with sampling every 200 generations. Convergence was reached after the average standard deviation of the posterior probability was below 0.01 and the value of the potential scale reduction factor reached 1.00. The clade assignment was based on the previous histoplasmosis ITS1–ITS2 nucleotide sequences data published by other studies conducted in Japan, Australia, Mexico, Brazil, Argentina, China, Thailand, and several European and African countries (Kasuga et al., 2003; Landaburu et al., 2014). *Blastomyces dermatitidis* (EF592163.1) that was isolated from USA was included in the phylogenetic analysis as an outgroup in order to improve bootstrap values.

## Results

### Clinical Cases of Epizootic Lymphangitis

**Figure 1:**
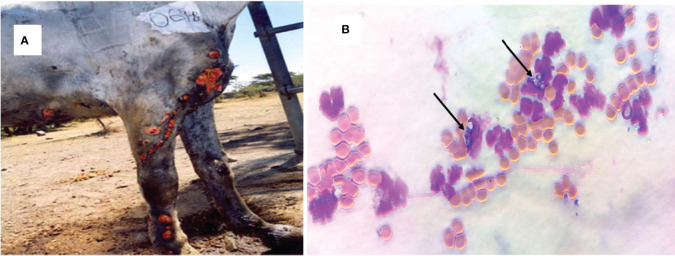
Clinical epizootic lymphangitis case of a carthorse and the yeast form of *H. capsulatum* var. *farciminosum* in central Ethiopia. **(A)** Granulomatous inflammation of the lymphatic vessels, the regional lymph nodes, and the skin of the fore limbs; as the disease progressed, the nodules ulcerated and formed ulcers. **(B)** Giemsa-stained yeast form of *H. capsulatum* var. *farciminosum* obtained from nodular aspirate and observed under microscope at ×100 with oil immersion. The yeast cells are shown by arrow; the purple cells are granulocytes while the orange cells are red blood cells.

The proportion of EL in the recruited horses was 25% (97/390) in central Ethiopia. However, only 67 horses that had unruptured nodular lesions were used for the aspiration of contents of nodules for culturing of *H. capsulatum*. The observed clinical lesions were characterized by a suppurative, nodular or ulcerating, spreading pyogranulomatous, multifocal dermatitis, and lymphangitis. As it is shown in **Figure 1A**, the disease affects the limbs, the chest wall, and the neck. In all cases, the lesions were nodular and granulomatous in character and spread locally by invasion and *via* the lymphatics. The yeast form *H. capsulatum* was demonstrated by staining the aspirates of the nodules using Giemsa stain in which it appears as centrally transparent oval shaped and located intracellularly in the macrophages or extracellularly in the nodular content (**Figure 1B**).

### Growth of Equine Histoplasma

**Figure 2:**
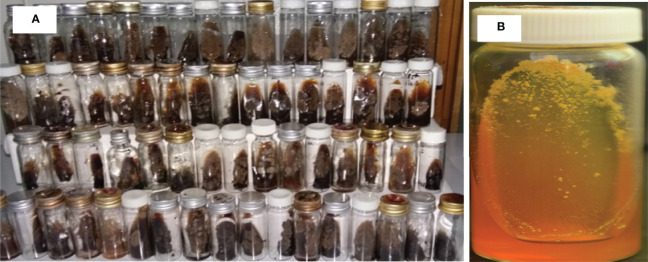
Mycelial and yeast colonies of *H. capsulatum* var. *farciminosum* isolated from epizootic lymphangitis cases of horses in central Ethiopia. **(A)** Mycelial colonies on 2.5% glycerol enriched Sabouraud Dextrose Agar grown at 26°C after 4–8 weeks of incubation. The slants show pure mycelial colonies that are abundant, gray/white in color at the early stage but becoming brown as they are aging. **(B)** Yeast colonies grown on the Brain Heart Infusion Agar at 37°C after incubation for 4–8 weeks. The yeast colonies are small in size and whitish gray in color.

The growth of mycelial colonies was confirmed in 60 horses. The colonies appeared between 4 and 8 weeks of incubation as gray-white at the early stage, but as the colonies were aging, they became brown in color (**Figure 2A**). The mycelial colonies were abundant and voracious in feeding, emptying the 10-ml Sabouraud Dextrose Agar within a few months. Similarly, the yeast form was grown on the Brain Heart Infusion Agar and the colonies were gray in color and relatively small in size (**Figure 2B**). In the present study, the mycelial colonies were harvested for the extraction of DNA and subsequent molecular activities.

### Identification of Histoplasma by PCR and Blasting of the Sequence Data

**Figure 3:**
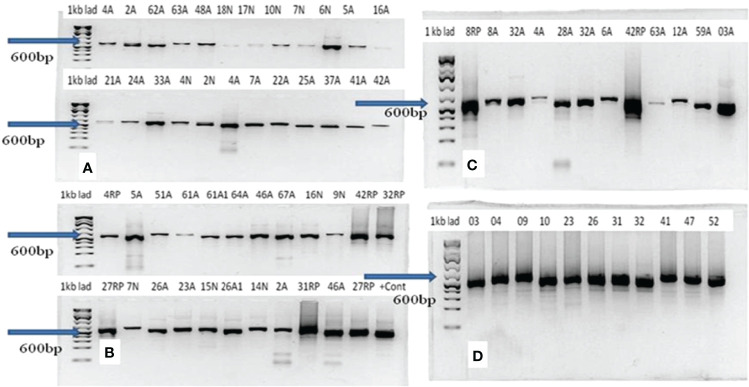
Result of gel electrophoresis of PCR products of *Histoplasma capsulatum* isolated from epizootic lymphangitis cases of horses in central Ethiopia. In each gel **(A–D)**, the left lane is the 1-kb ladder while the remaining 12 lanes (2–13) represent samples. The molecular weights of all PCR products were equal and were about 600 bp as indicated by arrows on each gel.

**Figure 4:**
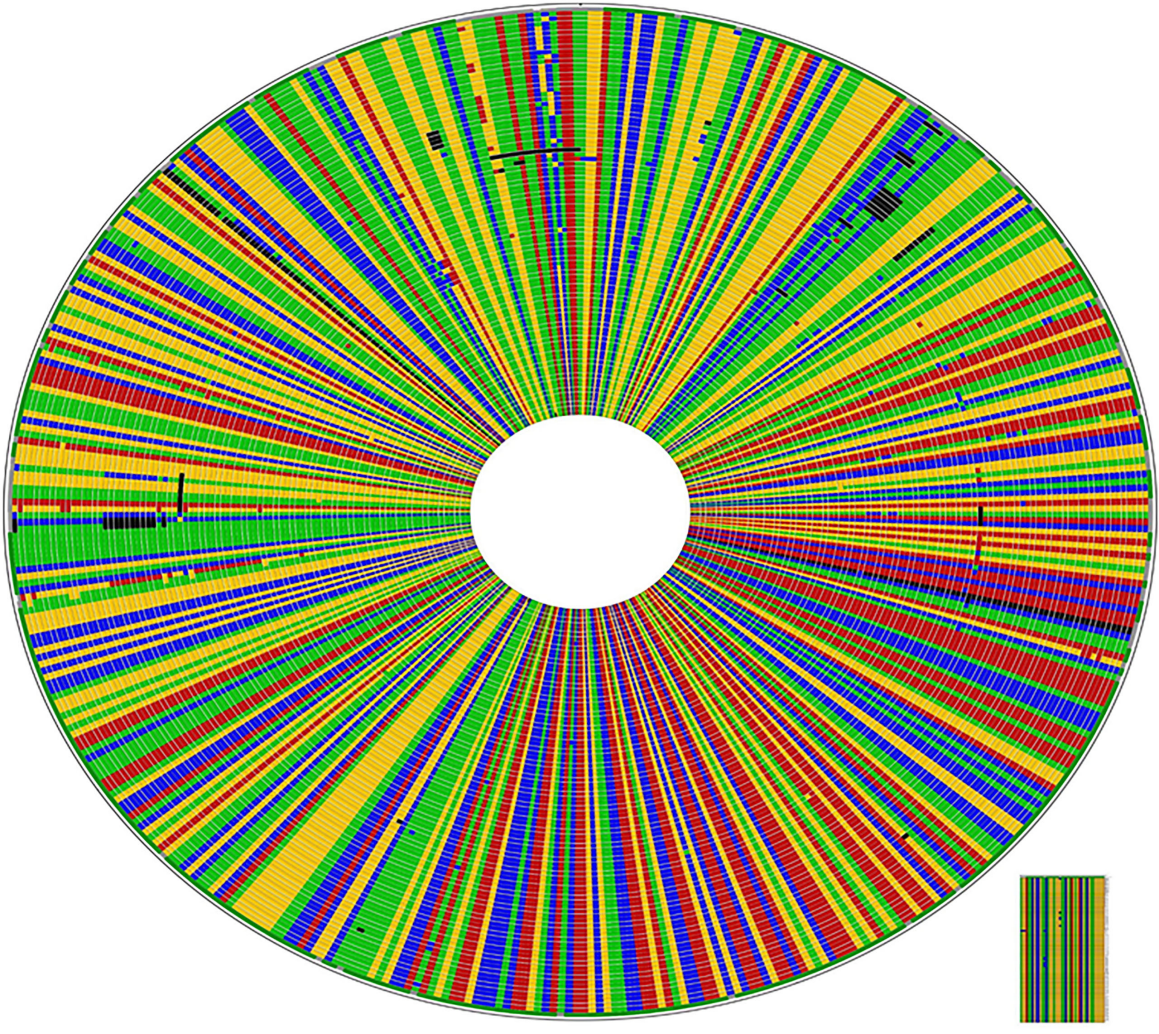
High rate of conservation amongst Ethiopian *Histoplasma capsulatum* in ITS1-5.8s-ITS2 region: CLUSTAL W alignment of the 92 isolates including the 29 generated from this study. The sequences different from the consensus sequences are highlighted by a different color; dark colors indicate gaps.

All the 60 culture-positive samples were also positive with PCR for *Histoplasma*. The molecular weights of the PCR products of all the 54 samples were similar and weigh about 600 bp (**Figures 3A–D**). The qualities and concentration of the 60 PCR products were assessed by further sequencing, but six PCR products failed to fulfill the required quality and concentration for library preparation. Thus, the library of 54 DNA samples was submitted for sequencing excluding the six PCR samples. BLAST analysis of these 54 isolates identified 29 *H. capsulatum* isolates and 14 isolates from other fungal genera (**Table 1**), while the remaining 11 samples were deemed insufficient for further downstream analysis. The alignment of the sequence data of the 29 Ethiopian isolates showed highly conserved ITS regions (**Figure 4**). There were a few gaps at the beginning of the sequences, and uneven sequence lengths were observed because of the deletions of sequences; the longest sequences have 653 bp while the shortest sequences have 553 bp.

**Table 1:** Histoplasma and other fungal genera identified by blasting the sequence data.

Name of genus	Number (%)	Remark
Aspergillus spp.	4(7.4%)	For the Fasta alignment, please refer to folder “Aspergillus”
Eurotium	3(5.6%	For the Fasta alignment, please refer to folder “Eurotium”
Penicillium	3(5.6%	For the Fasta alignment, please refer to folder “Penicillium”
Cladosporioides	4(7.4%)	For the Fasta alignment, please refer to folder “Cladosporioides”
Histoplasma	32(59.3%)	For the Fasta alignment, please refer to folder “Histoplasma”
Uncultured fungus	8(14.8%)	For the Fasta alignment, please refer to folder “uncultured Fungus clone”

### Phylogenetic Relationships Between the Ethiopian Isolates and Other Isolates

**Figure 5:**
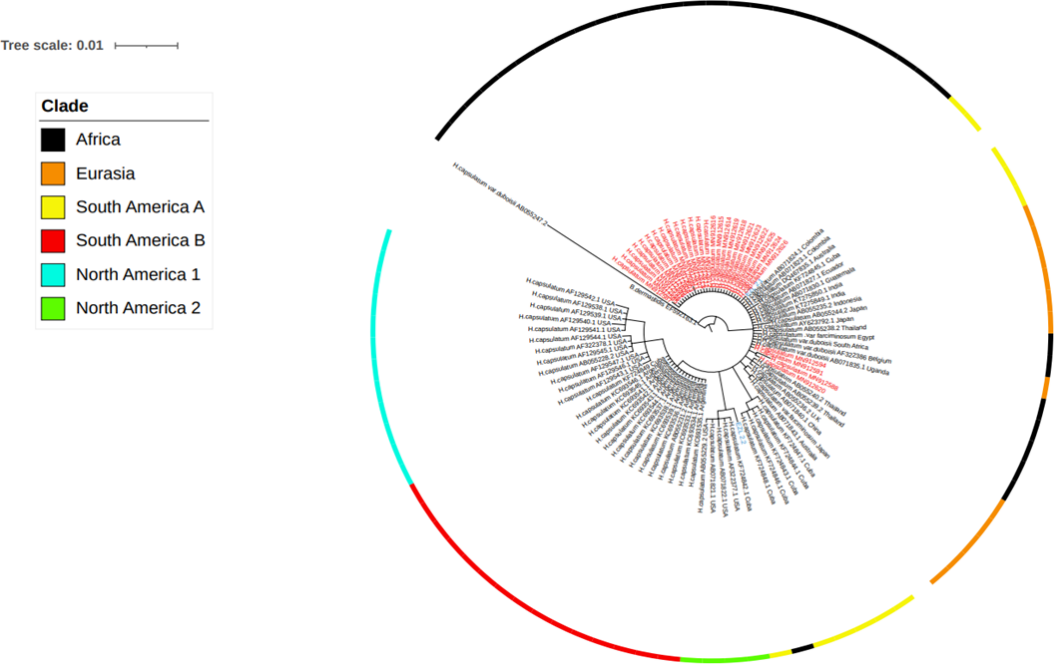
Evolutionary analysis of *Histoplasma capsulatum* isolated from Ethiopia and other countries by maximum likelihood method. The evolutionary history was inferred by using Bayesian inference with the Tamura-Nei model + gamma model. Clades are presented as color tips parallel to sample names which also indicate their countries of origin and NCBI accession number. Sample names written in red color font (Ethiopian) are those generated by the present study, while two Ethiopian isolates that are written in blue font color were generated by previous study in Ethiopia.

The sequence data of isolates from different countries were included for the phylogenetic analysis. The accession number and the origin of these isolates are presented in **Table 2**. The phylogenetic analysis identified six clades. These clades include the African, Eurasian, North American 1 and 2, and South American A and B. Almost all the Ethiopian isolates recovered by the present study were grouped under the African clade and were closely clustered with isolates from South American A and Eurasian clades (**Figure 5** and **Supplementary File**). Similarly, one Ethiopian of the two isolates reported from Ethiopia by a previous study and named as EZL 2.1 was also clustered with the new Ethiopian isolates, while the second isolate which was designated EZL 2.2 was further away from the Ethiopian isolates of this study. The Ethiopian isolates were clustered further from the clades of North America 1, North America 2, and South America B.

**Table 2:** Accession numbers of the *H. capsulatum* isolates included in the phylogenetic analysis and their countries of origin.

Accession number	Strain	Country of origin	Source	Clade
AF322378.1	*Histoplasma capsulatum*	USA	Dog	NAm_1_
AB055229.2	*Histoplasma capsulatum*	USA	Human	NAm_2_
AB071821.1	*Histoplasma capsulatum*	USA	Human	NAm_2_
AB071822.1	*Histoplasma capsulatum*	USA	Human	NAm_2_
AF129545.1	*Histoplasma capsulatum*	USA	Human	NAm_2_
AF129542.1	*Histoplasma capsulatum*	USA	Human	NAm_1_
AB055228.2	*Histoplasma capsulatum*	USA	Human	NAm_1_
AF129547.1	*Histoplasma capsulatum*	USA	Human	NAm_1_
AF129546.1	*Histoplasma capsulatum*	USA	Human	NAm_1_
AF129543.1	*Histoplasma capsulatum*	UK	Human	Eurasia
AF129544.1	*Histoplasma capsulatum*	Thailand	Human	Eurasia
AF129538.1	*Histoplasma capsulatum*	Thailand	Human	Eurasia
AF129539.1	*Histoplasma capsulatum*	Thailand	Human	Eurasia
AF129540.1	*Histoplasma capsulatum*	Japan	Human	Eurasia
AF129541.1	*Histoplasma capsulatum*	Japan	Human	Eurasia
AB055236.2	*Histoplasma capsulatum*	Indonesia	Human	Eurasia
AB055238.2	*Histoplasma capsulatum*	Thailand	Human	Eurasia
AB055239.2	*Histoplasma capsulatum*	Thailand	Human	Eurasia
AB055240.2	*Histoplasma capsulatum*	Thailand	Human	Eurasia
AY623792.1	*Histoplasma capsulatum*	Japan	Dog	Eurasia
AB055244.2	*Histoplasma capsulatum*	Japan	Human	Eurasia
AB055235.2	*Histoplasma capsulatum*	Indonesia	Human	Eurasia
AB071830.1	*Histoplasma capsulatum*	Guatemala	Human	SAm_A_
AB071827.1	*Histoplasma capsulatum*	Ecuador	Human	SAm_A_
AB071823.1	*Histoplasma capsulatum*	Colombia	Human	SAm_A_
AB071840.1	*Histoplasma capsulatum*	China	Human	Eurasia
AB071843.1	*Histoplasma capsulatum*	Australia	Human	Eurasia
KC693546.1	*Histoplasma capsulatum*	Argentina	Skin	SAm_B_
KC693545.1	*Histoplasma capsulatum*	Argentina	Larynx biopsy	SAm_B_
KC693541.1	*Histoplasma capsulatum*	Argentina	LRC HIV	SAm_B_
KC693542.1	*Histoplasma capsulatum*	Argentina	Skin HIV	SAm_B_
KC693543.1	*Histoplasma capsulatum*	Argentina	Blood culture HIV	SAm_B_
KC693544.1	*Histoplasma capsulatum*	Argentina	Oral mucosa HIV	SAm_B_
KC693537.1	*Histoplasma capsulatum*	Argentina	Skin HIV	SAm_B_
KC693538.1	*Histoplasma capsulatum*	Argentina	Skin	SAm_B_
KC693539.1	*Histoplasma capsulatum*	Argentina	Bone marrow HIV	SAm_B_
KC693536.1	*Histoplasma capsulatum*	Argentina	Blood culture HIV	SAm_B_
AB055231.2	*Histoplasma capsulatum*	Argentina	human	SAm_B_
KC693533.1	*Histoplasma capsulatum*	Argentina	Blood culture HIV	SAm_B_
KC693534.1	*Histoplasma capsulatum*	Argentina	Nasal mucosa	SAm_B_
KC693535.1	*Histoplasma capsulatum*	Argentina	Oral mucosa	SAm_B_
MN912626	*Histoplasma capsulatum*	Ethiopia (this study)	Horse	
MN912624	*Histoplasma capsulatum*	Ethiopia (this study)	Horse	
MN912625	*Histoplasma capsulatum*	Ethiopia (this study)	Horse	
MN912622	*Histoplasma capsulatum*	Ethiopia (this study)	Horse	
MN912623	*Histoplasma capsulatum*	Ethiopia (this study)	Horse	
MN912620	*Histoplasma capsulatum*	Ethiopia (this study)	Horse	
MN912621	*Histoplasma capsulatum*	Ethiopia (this study)	Horse	
MN912618	*Histoplasma capsulatum*	Ethiopia (this study)	Horse	
MN912619	*Histoplasma capsulatum*	Ethiopia (this study)	Horse	
MN912614	*Histoplasma capsulatum*	Ethiopia (this study)	Horse	
MN912615	*Histoplasma capsulatum*	Ethiopia (this study)	Horse	
MN912616	*Histoplasma capsulatum*	Ethiopia (this study)	Horse	
MN912617	*Histoplasma capsulatum*	Ethiopia (this study)	Horse	
MN912612	*Histoplasma capsulatum*	Ethiopia (this study)	Horse	
MN912613	*Histoplasma capsulatum*	Ethiopia (this study)	Horse	
MN912607	*Histoplasma capsulatum*	Ethiopia (this study)	Horse	
MN912611	*Histoplasma capsulatum*	Ethiopia (this study)	Horse	
MN912599	*Histoplasma capsulatum*	Ethiopia (this study)	Horse	
MN912603	*Histoplasma capsulatum*	Ethiopia (this study)	Horse	
MN912604	*Histoplasma capsulatum*	Ethiopia (this study)	Horse	
MN912605	*Histoplasma capsulatum*	Ethiopia (this study)	Horse	
MN912596	*Histoplasma capsulatum*	Ethiopia (this study)	Horse	
MN912597	*Histoplasma capsulatum*	Ethiopia (this study)	Horse	
MN912598	*Histoplasma capsulatum*	Ethiopia (this study)	Horse	
MN912595	*Histoplasma capsulatum*	Ethiopia (this study)	Horse	
MN912587	*Histoplasma capsulatum*	Ethiopia (this study)	Horse	
MN912588	*Histoplasma capsulatum*	Ethiopia (this study)	Horse	
MN912591	*Histoplasma capsulatum*	Ethiopia (this study)	Horse	
MN912594	*Histoplasma capsulatum*	Ethiopia (this study)	Horse	
EF592163.1	*Blastomyces dermatitidis*			

NAm_1_, North America 1 clade; NAm_2_, North America 2 clade; SAm_A_, South America A clade; SAm_B_, South America B clade.

## Discussion

In the present study, *H. capsulatum* was isolated from 60 EL cases of horses in central Ethiopia, of which the DNA samples of the isolates from 54 could qualify for further molecular analysis using sequencing of the ITS region. BLAST analysis of 54-sequence data identified 29 *H. capsulatum* while 14 were identified as other fungal genera including Aspergillus, Eurotium, Penicillium, and Cladosporioides. However, the remaining 11 DNA samples were not deemed of sufficient length to continue downstream analysis and hence were removed. The source of the other fungal genera could not be identified as the cultures were pure and had similar colonies. In addition, as it can be observed from the gels, the PCR products of each sample had a clear single band. Hence, it could be difficult to explain the origin of these fungal genera.

Since the ITS region is a universal to fungal species, it was thus difficult to classify the varieties of *Histoplasma capsulatum* into *H. capsulatum* var. *capsulatum*, *H. capsulatum* var. *farciminosum*, or *H. capsulatum* var. *duboisii*. Therefore, in this study, phylogenetic analysis could group the isolates at the species level of *Histoplasma capsulatum* to evaluate the relationship among the isolates of Histoplasma from different countries. The phylogenetic analysis grouped the global isolates into African, Eurasian, North American 1 and 2, and South American A and B. All of the newly isolated Ethiopian Histoplasma isolates were grouped with the South American A and Eurasian clades. However, they were clustered distantly furthest away from the North American 1 and 2 as well as South American B clades.

In addition to Ethiopia, the African clade was isolated from Egypt, Uganda, and South Africa. This clade is closely related with the Latin American A clade. In addition, the African clade is related to the Eurasian clade. The Eurasian clade was isolated from Europe (Belgium) and Asian countries (Thailand, Japan, Indonesia, India, and China). The South American A clade was originally isolated from the Latin American countries including Cuba, Colombia, Ecuador, and Guatemala covering a broader geographic coverage. The complex genetic population structure of the South American A clade was reported earlier (Teixeira M de et al., 2016). Unlike the South American A clade, the South American B clade is limited to isolates from Argentina, as it can observed in the phylogenetic tree of this study. The South American A and B clades were reported by Kasuga and coworkers (Kasuga et al., 2003); however, Sepúlveda and coworkers reported only the South American A clade (Sepúlveda et al., 2017).

Previous studies have reported the North American 1 and North American 2 clades (Kasuga et al., 2003; Sepúlveda et al., 2017). The North American 2 clade was isolated from humans in USA and limited to USA so far, as it has not been reported from other countries. Both the North American 1 and 2 clades are not related to the Ethiopian isolates. This suggests the less likely chance of transmission of *Histoplasma* organisms between Ethiopia and USA. In addition to the present isolates, EZL 2.1 and EZL 2.2 were reported from Ethiopia earlier by other researchers (Scantlebury et al., 2016). In the phylogenetic tree of the present study, EZL 2.1 was grouped closely with the isolates of the present study. However, EZL 2.2 was grouped further from the other Ethiopian isolates.

Different authors classified the clades of *Histoplasma capsulatum* based on different markers. Kasuga and coauthors reported eight different clades using the phylogenetic analysis of 137 isolates based on the DNA sequence variation in four independent protein-coding genes (Kasuga et al., 2003). The authors identified the North American 1 and 2, Latin American A and B, Australian, Netherlands (Indonesia)?, Eurasian, and African clades. As reported by these authors, seven of the eight clades can be considered as recognized phylogenetic species while the Eurasian clade could be classified under the Latin American A clade. A more recent study proposed six additional phylogenetic species of *Histoplasma* within Latin American isolates using 234 isolates by increasing the taxon sampling and using different phylogenetic and population genetic methods (Teixeira M de et al., 2016). Thus, there are different ways of classifying the isolates of *H. capsulatum* by different authors, which warrants reaching on a consensus single method that can be used for all researchers working on *H. capsulatum*.

Previous studies classified *H. capsulatum* into three varieties (Kasuga et al., 1999). According to this study, *H. capsulatum* var. *capsulatum* causes histoplasmosis in humans primarily affecting the lungs. Furthermore, *H. capsulatum* var. *capsulatum* was also isolated from domestic cats with severe and disseminated mycosis in the lungs (Panciera, 1969; Arunmozhi Balajee et al., 2013). Moreover, histoplasmosis due to *H. capsulatum* var. *capsulatum* was reported in dogs in USA (Brömel and Sykes, 2005). Although *H. capsulatum* var. *capsulatum* was reported from about 60 countries from all continents (Ajello, 1988), it is highly prevalent in the North America and Latin America (Kwon-Chung et al., 1992). There are two clades of *H. capsulatum* var. *capsulatum* in North America which differ in growth phenotypes, restriction fragment length polymorphisms of mitochondria, and genomic DNA (Vincent et al., 1986; Spitzer et al., 1989). The North American 1 isolates were isolated from HIV patients while the North American 2 isolates were isolated from both individuals infected with HIV and individuals not infected with HIV (Spitzer et al., 1990).

*H. capsulatum* var. *duboisii* is considered to be the causative agent of African histoplasmosis in humans that is characterized by cutaneous, subcutaneous, and osseous lesions (Stelzner and Rippon, 1990), while *H. capsulatum* var. *farciminosum* causes subcutaneous and ulcerated lesions in the skin of horses and mules (Stelzner and Rippon, 1990). Additionally, *H. capsulatum* var. *farciminosum* causes similar lesions in dogs (Ueda et al., 2003). Animal histoplasmosis is widespread throughout Europe, North America, India, and South Asia (Kwon-Chung et al., 1992). In addition, *H*. *capsulatum* var. *farciminosum* was isolated from soil in caves infested with bats in Israel (Ajello et al., 1977), suggesting the possibility of its transmission by bats which can excrete the fungus through feces or other secretions that can contaminate the soil, thereby facilitating its further transmission to equines. Additionally, disseminated histoplasmosis was diagnosed in domestic cats and in wild badgers (*Meles meles*) in Austria (Klang et al., 2013) and in Germany (Eisenberg et al., 2013), respectively, suggesting the importance of these species of animals in the epidemiology of histoplasmosis. The morphology of the yeast cells of *H. capsulatum* var. *farciminosum* resembles that of *H. capsulatum* var. *capsulatum* (Panciera, 1969).

This study is the first study in sequencing a relatively large number of *H*. *capsulatum* var. *farciminosum* isolated from EL cases of horses. In addition, the sequences of Ethiopian isolates were compared with the sequence data of *H. capsulatum* isolates that were retrieved from GenBank. This effort can be considered as the strength of this study. However, the weakness of the study is the use of the internal transcribed spacer (ITS) region of rRNA genes, which is a universal gene in fungal organisms.

## Conclusion

In conclusion, according to the phylogenetic analysis of the sequences of the ITS region of rRNA genes of *Histoplasma*, the Ethiopian isolates were closely related to the South American A and Eurasian clades while they were distantly clustered further away from the North American 1 and 2 and South American B clades.

## Data Availability Statement

The datasets presented in this study can be found in online repositories. The names of the repository/repositories and accession number(s) can be found as follows: https://www.ncbi.nlm.nih.gov/nuccore/MN912587.1/, MN912587-MN912626.

## Ethics Statement

Ethical review and approval were not required for the animal study because the screening cases and sample collection were performed as part of the clinical examination and treatment of epizootic cases of horses in Woliso Town, central Ethiopia. However, consent was obtained from owners for the collection of pus samples. Written informed consent was obtained from the owners for the participation of their animals in this study.

## Author Contributions

GA and TS designed the study. AZ and AMK contributed in the analysis of the data. MK and FS supervised the preparation of library for sequencing and facilitated the sequencing. RA and MGA contributed in the field sample collection and culturing of the samples. GA was involved in the field sample collection, fungal isolation, and preparation of the library. GA drafted the manuscript while all the other authors edited the manuscript. All authors contributed to the article and approved the submitted version.

## Funding

The research was financially supported by the National Human Genomic Institute, National Institute of Health (Reference Number U01HG007472).

## Conflict of Interest

The authors declare that the research was conducted in the absence of any commercial or financial relationships that could be construed as a potential conflict of interest.

## Publisher’s Note

All claims expressed in this article are solely those of the authors and do not necessarily represent those of their affiliated organizations, or those of the publisher, the editors and the reviewers. Any product that may be evaluated in this article, or claim that may be made by its manufacturer, is not guaranteed or endorsed by the publisher.
