# Supplementary figures and images for: Equine Histoplasmosis in Ethiopia: Phylogenetic Analysis by Sequencing of the Internal Transcribed Spacer Region of rRNA Genes
Source: Front Cell Infect Microbiol. 2022 Jul 8;12:789157. doi: 10.3389/fcimb.2022.789157 (PMC9330904; doi:10.3389/fcimb.2022.789157)

Tree scale: 0.01

Clade

Africa

Eurasia

South America A

South America B

North America 1

North America 2

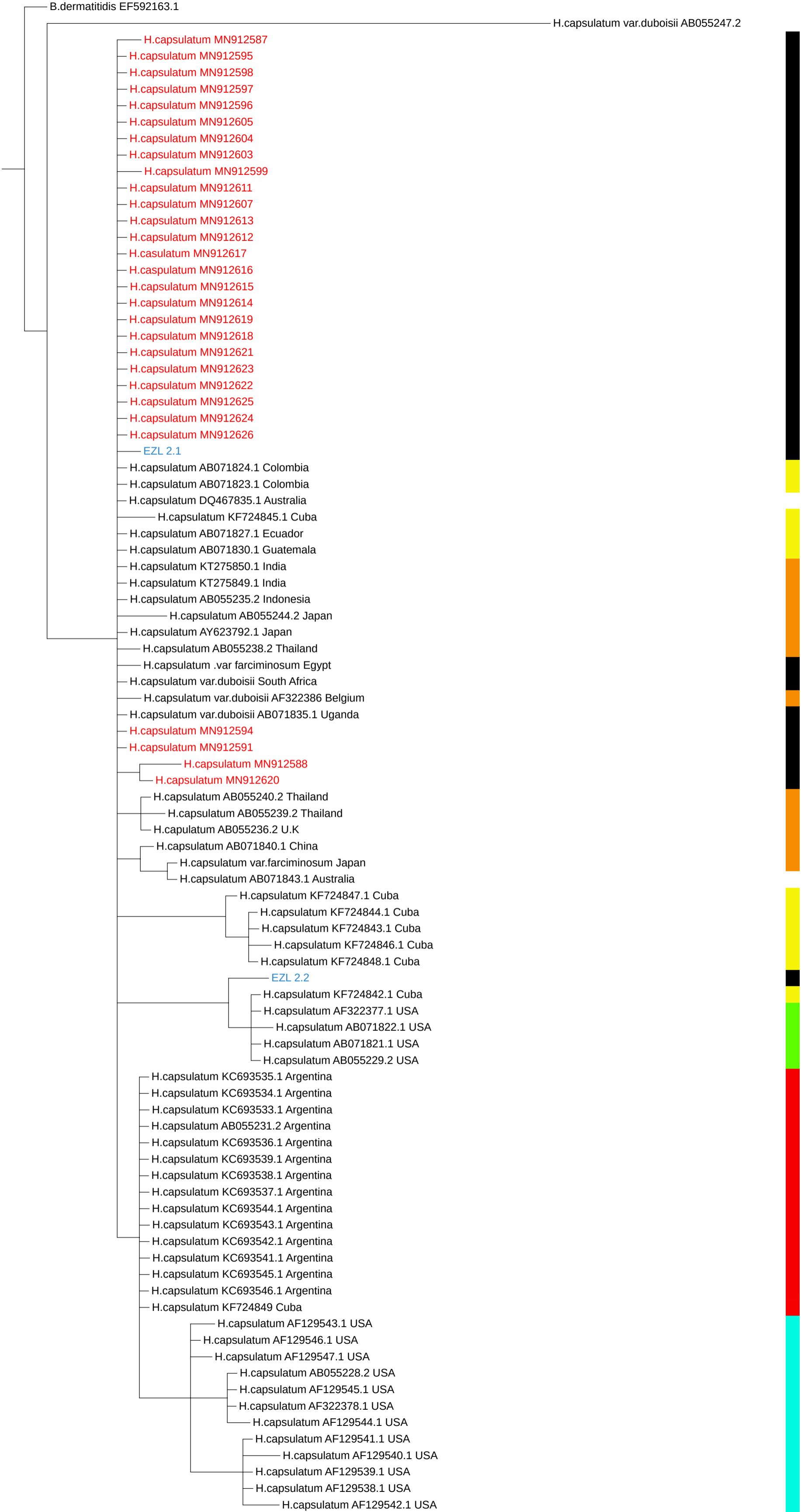

Supplement: Supplementary file 1 [file Image_1.pdf]
